# Tinnitus Severity Is Reduced with Reduction of Depressive Mood – a Prospective Population Study in Sweden

**DOI:** 10.1371/journal.pone.0037733

**Published:** 2012-05-22

**Authors:** Sylvie Hébert, Barbara Canlon, Dan Hasson, Linda L. Magnusson Hanson, Hugo Westerlund, Töres Theorell

**Affiliations:** 1 École d’orthophonie et d’audiologie, Faculté de médecine, Université de Montréal, BRAMS, International Laboratory for Brain, Music, and Sound Research, and Centre de recherche de l’Institut universitaire de gériatrie de Montréal, Montréal, Québec, Canada; 2 Stress Research Institute, Stockholm University, Stockholm, Sweden; 3 Department of Public Health, Karolinska Institute, Stockholm, Sweden; 4 Department of Physiology and Pharmacology, Karolinska Institute, Stockholm, Sweden; University of Regensburg, Germany

## Abstract

Tinnitus, the perception of sound without external source, is a highly prevalent public health problem with about 8% of the population having frequently occurring tinnitus, and about 1–2% experiencing significant distress from it. Population studies, as well as studies on self-selected samples, have reported poor psychological well-being in individuals with tinnitus. However, no study has examined the long-term co-variation between mood and tinnitus prevalence or tinnitus severity. In this study, the relationship between depression and tinnitus prevalence and severity over a 2-year period was examined in a representative sample of the general Swedish working population. Results show that a decrease in depression is associated with a decrease in tinnitus prevalence, and even more markedly with tinnitus severity. Hearing loss was a more potent predictor than depression for tinnitus prevalence, but was a weaker predictor than depression for tinnitus severity. In addition, there were sex differences for tinnitus prevalence, but not for tinnitus severity. This study shows a direct and long-term association between tinnitus severity and depression.

## Introduction

Tinnitus is an increasing public health problem in modern societies. Its prevalence is estimated to be about 25% in the general population [1] and about 8% for frequently occurring tinnitus [2]. Tinnitus prevalence is increasing in young adults [3] and it is presumed to be related with exposure to high volume music in portable devices as well as to noisy environments [4]. Another important etiological factor among older men is a history of exposure to noise in heavy industry and military operations [2], [5], [6]. Indeed, the most important predictor of tinnitus is hearing loss, especially at high frequencies, and recent estimates of the prevalence of hearing loss range from 2% to 93% depending on gender and age, with an overall rate of 31% for the whole US population [5]. Hearing loss has been found to predict tinnitus and to a lesser extent tinnitus severity [7], [8]. Tinnitus severity indicates the extent to which the individual is bothered, upset or worried by the tinnitus. Hearing pathology has been linked to tinnitus even when there is no measurable hearing loss in the audiogram [9], [10].

During recent years [11], [12], the relationship between psychological well-being and tinnitus has been emphasized. There are many symptoms of long-lasting stress that are associated with tinnitus. A recent population study reported that emotional exhaustion is a strong predictor of tinnitus severity [7]. However cross-sectional studies do not allow ascertaining the causality of such relationships, or how well-being co-varies over time with tinnitus severity. Tinnitus may cause distress, giving rise to a deterioration of psychological well-being. The reverse relationship could also prevail [13]. Feelings of distress, associated with tinnitus, may increase during periods of poor psychological well-being. Population studies have found associations between tinnitus and generalized anxiety disorders [2] and depressive symptoms [14]. However, individuals with tinnitus may have depressive symptoms but not meet the clinical diagnosis of major depressive disorder [2]. Indirect evidence suggests that reducing tinnitus-related distress also reduces psychopathological symptoms. A recently published Cochrane review [15] came to the conclusion that cognitive behavioral therapy (CBT) is not likely to reduce the subjective loudness of tinnitus using a visual analogue scale (VAS). The value of using these scale types for tinnitus severity assessment has been questioned [16]. However, in six randomized trials, CBT has been shown to improve tinnitus-related distress scores and in five other similar studies to improve the quality of life in tinnitus patients. Another recent systematic review, involving nine additional trials and 806 patients, demonstrated that CBT had a further positive effect on mood scores and stress reduction [17]. Tinnitus-related distress was still reduced at 18 months follow-up, although effect sizes decreased over time. Mood was not assessed beyond 12 months, and effect sizes varied substantially. In addition, it was not possible to know whether the positive effects of the therapy were due to a reduction in anxiety and/or depression symptoms since depression and anxiety measures were combined into a single mood construct. Nevertheless, there is some evidence that tinnitus severity and mood co-vary, at least over the short-term, although direct relationships or long-term assessments have not yet been reported.

The focus of the present study is to investigate the relationship between depression and tinnitus prevalence and severity. More specifically, the study is longitudinal and conducted on a representative sample of the general Swedish working population. It is therefore possible to examine the relationship between a two-year change in depression score and a change in tinnitus or tinnitus severity during the same period. Our hypothesis was that an improvement in depression symptoms would be associated with an improvement in tinnitus prevalence and severity, and vice versa.

## Methods

### Population

Respondents from the 2003 and 2005 Swedish Work Environment Survey (SWES) conducted biennially by Statistics Sweden (SCB) were invited to enroll in the Swedish Longitudinal Occupational Survey of Health (SLOSH) [18], which was initiated by the Stress Research Institute in 2006. These are subsamples of gainfully employed people and stratified by county, sex, citizenship, and inferred employment status. Data collection was conducted in April 2008. A total of 18,734 individuals were mailed self-completion questionnaires in 2008, out of which 11,441 (61%) responded. However, the present study use only 9,756 (52% of the sample) who were working at the time of the survey (the rest being retired, unemployed or otherwise working less than 30% of full-time). In 2010 the total response rate was 57%. Table 1 shows the numbers of subjects responding to different parts of the present study in 2006 and 2008. For the final prospective analyses, 6,215 individuals who had complete data from both 2008 and 2010 for all the variables in this study were included in the analyses:

**Table 1:** Characteristics of male and female participants.

	Men				Women			
	2008		2010		2008		2010	
	M (± SD)	n	M (± SD)	n	M (± SD)	n	M (± SD)	n
**Age (20–70)**	49.8 (11.5)	5,086	53.3 (11.1)	3,778	48.7 (11.7)	6,355	51.8 (11.3)	4,993
**Income (elog)**	5.74 (0.50)	5,061	5.84 (0.49)	3,762	5.47 (0.49)	6,331	5.55 (0.52)	4,971
**Tinnitus**	1.58 (0.98)	4,969	1.63 (1.01)	3,735	1.35 (0.75)	6,234	1.36 (0.77)	4,919
**Tinnitus severity**	2.14 (0.82)	1,397	2.07 (0.85)	1,348	2.15 (0.78)	1,179	2.02 (0.81)	1,223
**Depression symptoms**	11.08 (4.96)	4,365	10.51 (4.66)	2,908	12.03 (5.45)	5,171	11.88 (5.37)	3,821
**Hearing loss**	0.65 (0.73)	4,354	0.72 (0.78)	3,699	0.56 (0.70)	5,164	0.58 (0.73)	4,886

#### Socioeconomic (SES) status in 2008 (SEK/year)

The variable corresponding to SES was annual income (taken from the tax registry). This variable was markedly skewed and was subjected to n-logarithmic transformation that provided a perfect normal distribution (range after transformation 0–8.23). Adjustments were also made for age and sex.

#### Tinnitus in 2008 and 2010 (range 1–4)

The single question for determining tinnitus was: Have you during the most recent time experienced sound in any of the ears, without there being an external source (so-called tinnitus) lasting more than five minutes? (No, Yes sometimes, Yes often, Yes all the time). This variable was slightly skewed (skewness 1.94 in 2008 and 1.90 in 2010). The questions about tinnitus were adapted from Davis [19] and Palmer et al. [20].

#### Tinnitus severity in 2008 and 2010 (range 1–4)

The single item for determining tinnitus severity was: How much do you feel that the tinnitus sounds worry, bother or upset you? (Not at all, A little, Moderately, Severely). The questions about tinnitus were adapted from Davis [19]and Palmer et al. [20]. The tinnitus severity item was answered only by those who reported that they experienced tinnitus (skewness 0.36 in 2008 and 0.45 in 2010).

#### Depressive symptoms in 2008 and 2010 (range 6–30)

Depressive symptoms were measured with a brief subscale from the Hopkins Symptom Checklist [21]. This particular version was based upon clinical validity, and focused on the six items corresponding to the Hamilton Depression sub-scale HAM-D_6_ [22]. Feeling blue? Feeling no interest in things? Feeling lethargy or low in energy? Worrying too much about things? Blaming yourself for things? Feeling everything is an effort? (last week; 0 = not at all to 4 = extremely). A sum score was used and it was nearly normally distributed (skewness 1.14 in 2008 and 1.19 in 2010).

#### Subjective hearing loss in 2008 and 2010 (range 0–3)

Subjective hearing loss was assessed with the question: How difficult is it for you to (without hearing aid) hear what is said in a conversation between several persons? (0 = not difficult at all to 3 = very difficult). In this study, *subjective* hearing loss reflects difficulties in communicating. The question about hearing loss was derived from Statistics Sweden and has been used in several population studies (see for instance [1] 2010).

### Statistical Analyses

Paired t-tests were conducted in order to assess possible changes in outcome variables between 2008 and 2010. Since all variables, explanatory as well as outcome, were close to normally distributed, multiple linear regressions were computed with all explanatory variables entered in one single step. Changes in depression and hearing loss were obtained by subtracting the 2008 scores from the respective depression and hearing loss 2010 scores. All the study variables have skewness estimates between −1.0 and 1.2 which is regarded as acceptably close to normal distributions with one important exception, the tinnitus variable which has skewness 1.9 for both years, which could possibly give rise to error. However, we also tested an ordinal multiple logistic regression using an ordinal version of tinnitus in 2008 as explanatory and similarly an ordinal version of tinnitus 2010 as dependent variables. This analysis showed that the same variables came out as significant independent predictors for tinnitus in 2010 as in the multiple linear regression. For the tinnitus severity variable there was no such problem (skewness 0.36 in 2008 and 0.45 in 2010), and for simplicity we therefore use the same statistical model for both outcomes. We chose linear regression since this seems to provide an adequate albeit not perfect solution.

We first regressed tinnitus score in 2010 on sex, age, income (n log transformed), depression score in 2008, change in depression score from 2008 to 2010, hearing loss in 2008, change in hearing loss from 2008 to 2010, and tinnitus score in 2008. Restricting the analysis to only those who reported tinnitus in 2008, we then regressed tinnitus severity in 2010 on the same explanatory variables, except that tinnitus score in 2008 was replaced by tinnitus severity in 2008. The variable income was markedly skewed to the right (which is typical of this variable in population studies), with a small number of participants with a very high income. Since these participants could give rise to distortion in analyses including income this variable was transformed logarithmically.

The software JMP®, Version 9 (SAS Institute Inc., Cary, NC) was used for all statistical analyses. Significance level was set at p<0.05.

The Regional Research Ethics Committee in Stockholm (Ref no 2006/158-31) has approved the study. There was written consent from every participant and the committee perused the conditions of the study after which they gave their consent. No children or relatives participated.

**Table 2:** Tinnitus score in 2010 as dependent variable in relation to predictors.

	Standardized β	B	SEM(B)	t	p	Chi square (p)
**Intercept**		0.801	0.113	7.06	<0.0001	
**Sex (m = 1, f = 2)**	−0.0315	−0.056	0.015	3.84	0.0001	12.80 (0.0003)
**Age (10 y)**	−0.0107	−0.008	0.007	1.10	0.27	2.65 (0.104)
**Income 2008 (elog)**	0.0025	0.004	0.016	0.26	0.79	0.07 (0.791)
**Depressive symptoms 2008**	0.0214	0.004	0.002	2.27	0.023	13.41 (0.0002)
**Change in depressive symptoms (2010–2008)**	0.0421	0.008	0.002	4.67	<0.0001	29.23 (0.0001)
**Tinnitus score 2008**	0.7451	0.752	0.008	90.67	<0.0001	2982.96 (0.0001)
**Hearing loss 2008**	0.1088	0.109	0.011	9.66	<0.0001	79.87 (0.0001)
**Change in hearing loss (2010–2008)**	0.0577	0.088	0.013	6.88	<0.0001	36.48 (0.0001)

Multiple linear regression (n = 6,095).

Adjusted r^2^ was 62.8%. Chi square and p values in the last column are obtained from ordinal logistic regression analysis.

(df = 3 for tinnitus score in 2008 and df = 1 for all other explanatory variables).

## Results

The characteristics of the participants are presented in Table 1, which is based upon participants in each wave separately. All numbers (n) of respondents for each variable (divided into men and women) are given in the table. The depression score decreased significantly in the population as a whole between 2008 and 2010 (paired samples t-test, *t* = 3.91, *p* = 0.0001, N = 6,340). The tinnitus score, on the other hand, did not change significantly. During the two years there was a significant increase in the mean value for the self-rated hearing loss (*t* = 2.13, *p* = 0.034, N = 7,173).

**Table 3:** Tinnitus severity score in 2010 as dependent variable in relation to predictors.

	Standardized β	B	SEM(B)	t	p	Chi square (p)
**Intercept**		1.623	0.319	5.09	<0.0001	
**Sex (m = 1, f = 2)**	−0.0118	−0.020	0.039	0.51	0.61	0.218 (0.641)
**Age (10 y)**	−0.0285	0.020	0.021	0.93	0.63	2.197 (0.138)
**Income 2008 (elog)**	−0.0288	−0.047	0.047	0.99	0.98	1.021 (0.312)
**Depressive symptoms 2008**	0.0848	0.013	0.004	3.22	0.005	8.876 (0.003)
**Change in depressive symptoms (2010–2008)**	0.1324	0.023	0.004	5.24	<0.0001	27.138 (0.0001)
**Tinnitus severity score 2008**	0.5027	0.523	0.025	20.74	<0.0001	385.95 (0.0001)
**Hearing loss 2008**	0.0406	0.196	0.026	7.59	<0.0001	144.874 (0.0001)
**Change in hearing loss (2010–2008)**	0.1030	0.148	0.032	4.66	<0.0001	17.698 (0.0001)

Multiple linear regression (n = 1,233). Adjusted r^2^ was 62.5%. Chi square and p values in the last column are obtained from ordinal logistic regression analysis (df = 3 for tinnitus score in 2008 and df = 1 for all other explanatory variables).

### Predictors of Tinnitus

Table 2 shows the results of a multiple linear regression analysis with tinnitus in 2010 as the dependent variable. The independent variables were: age, gender (m = 1, f = 2), income, tinnitus in 2008, depressive symptoms in 2008 and change in depressive symptoms from 2008 to 2010 as well as hearing loss in 2008 and change in hearing loss 2008–2010. The results demonstrated that sex (men have a higher prevalence of tinnitus) as well as baseline (2008) levels of tinnitus, depressive symptoms and hearing loss as well as change in depressive symptoms and hearing loss (2008–2010) were all statistically significant predictors of tinnitus in 2010. Age and income had no significant explanatory value. Analyses were then performed with men and women separately, yielding similar results to the combined model. The exception was that the predictive power of depressive symptom level in 2008 was statistically significant only in men (*p* = 0.039) but not in women (*p* = 0.137). Since all other factors that were highly predictive in the combined model were sex-independent (all *p*<0.002), we therefore present the results from the combined model. This model described 63% of the explained variance.

Table 3 is limited to participants with tinnitus and depicts the results of multiple regression analysis with tinnitus severity in 2010 as the dependent variable. The independent variables were: age, sex, income, tinnitus severity, depressive symptoms and hearing loss in 2008 and change in depressive symptoms and hearing loss (2008–2010). The results showed that tinnitus severity, depressive symptoms and hearing loss in 2008 as well as changes in depressive symptoms and hearing loss (2008–2010) were statistically significant predictors of tinnitus severity in 2010. Sex, age and income had no statistically significant explanatory value. Analyses were also performed with men and women separately. The results were very similar to the combined model, with the only observation that the predictive power of depressive symptom level in 2008 reached statistical significance only in women (*p* = 0.002) but not in men (*p* = 0.144). All factors that were highly significant predictors in the combined model were also predictive for women and men separately (all *p*s<0.001). Therefore we present the whole model, which describes 63% of the explained variance.

## Discussion

The main finding of the present study is that, within a random sample of Swedish workers, depression co-varies with tinnitus prevalence and tinnitus severity over time. That is, a decrease in depression is associated with a decrease in tinnitus prevalence, and even more markedly with severity, over a two-year period. Hearing loss and change in hearing loss are stronger predictors than depressive symptoms and change in depressive symptoms for *tinnitus prevalence*. However, hearing loss is a much less important predictor than depressive symptoms for *tinnitus severity*. To our knowledge, this is the first study to show a direct and long-term association between tinnitus severity and depression.

Langguth and colleagues [23] have recently argued that there are common pathways in the pathophysiology of tinnitus (especially tinnitus-related distress) and depression. Therefore, it is unlikely that these disorders are co-morbid only by chance and that depression is a direct reaction to tinnitus. However, depressive symptoms will make the tinnitus-related handicap more pronounced. In addition, in both depression and tinnitus, personality factors such as neuroticism and traits such as anxiety can also be predisposing factors [24], [25].

Tinnitus patients may not present signs of clinical depression, but rather a number of distress-related symptoms. Indeed, Ooms et al [26] found that the somatic depressive subscale of the Beck Depression Inventory II (BDI-II) [27] (not including the affective or cognitive subscales) predicted tinnitus severity. These authors challenged the idea that tinnitus is related to depression and instead proposed that the relationship between tinnitus handicap and depressive symptoms is a result of content overlap between the questionnaires BDI-II and the Tinnitus handicap inventory (THI). In our study, however, tinnitus severity and depression scores did not overlap. Tinnitus-related distress was assessed by a question related to somatic problems (i.e., How much do you feel that the tinnitus sounds worry, bother or upset you?), whereas depressive symptoms were assessed by questions mixing affective (e.g., Feeling blue? Feeling no interest in things? Blaming yourself for things) and somatic (e.g., Feeling lethargy or low in energy? Feeling everything is an effort?) items. Our study therefore does not support the idea that the association between depression and tinnitus severity is an artifact, but rather associated disorders that share core symptomatology or stem from a common etiology.

A recent study on 755 normal hearing individuals showed that tinnitus debut in older ages was more distressful than when the incidence occurred in younger ages [28]. Our population study could not corroborate this finding because data of the debut of tinnitus onset were not available. Furthermore, an effect of age on tinnitus prevalence or severity was not found. One explanation for this might be that our population was on the average below 50 years of age in 2008, and that the older age groups (>50 years), where prevalence rates of tinnitus typically increase with presbyacusis, were less represented here than in other studies.

Finally, another interesting finding of the present study was that sex predicted tinnitus prevalence, conditional on a number of other factors, but sex did not predict tinnitus severity. This finding is compatible with a recent population study showing a higher prevalence of frequent tinnitus in men than in women [2], and the reasons for this may be due to the higher prevalence of hearing loss in men compared to women. In contrast, hearing loss has a much less important role in tinnitus severity. For both men and women, the changes in depressive symptoms were predictive of tinnitus severity.

It is clear from our study that changes in depression and tinnitus scores are associated with each other and may be due to co-morbidity. It is important to note that this is a population study and not a self-selected sample of tinnitus or depressive patients. Therefore, the results can be broadly generalized on a population level. Future studies are needed to assess possible clinical relevance on an individual level. This study suggests that depressive symptoms should be assessed in tinnitus patients and vice versa. While it may be more common to assess depressive symptoms in tinnitus patients, assessing tinnitus in depressive patients is uncommon. Finally, this study highlights the importance of co-morbidity and its detrimental impact on tinnitus severity. Since the sound of tinnitus cannot be cured, our findings suggest that treating depressive symptoms, a significant tinnitus co-morbidity, should lead to a better quality of life by decreasing both depressive and tinnitus severity symptoms. Given the high prevalence of tinnitus, our study therefore is potentially a meaningful contribution to public health.
